# Topically Applied Biopolymer-Based Tri-Layered Hierarchically Structured Nanofibrous Scaffold with a Self-Pumping Effect for Accelerated Full-Thickness Wound Healing in a Rat Model

**DOI:** 10.3390/pharmaceutics15051518

**Published:** 2023-05-17

**Authors:** Kholoud H. Hamza, Ahmed A. El-Shanshory, Mona M. Agwa, Mohamed I. Abo-Alkasem, Esmail M. El-Fakharany, Abdallah S. Abdelsattar, Ali A. El-Bardan, Taher S. Kassem, Xiumei Mo, Hesham M. A. Soliman

**Affiliations:** 1 Department of Chemistry, Faculty of Science, Alexandria University, P.O. Box 426, Alexandria 21321, Egypt; kholoudhamza135@gmail.com (K.H.H.); alielbardan@yahoo.com (A.A.E.-B.); taherkasem@gmail.com (T.S.K.); 2 Composites and Nanostructured Materials Research Department, Advanced Technology and New Materials Research Institute (ATNMRI), City of Scientific Research and Technological Applications (SRTA-City), New Borg Al-Arab, Alexandria 21934, Egypt; h.soliman@srtacity.sci.eg; 3 Department of Chemistry of Natural and Microbial Products, Pharmaceutical and Drug Industries Research Institute, National Research Centre, Dokki, Giza 12622, Egypt; alkasem88@yahoo.com; 4 Protein Research Department, Genetic Engineering and Biotechnology Research Institute (GEBRI), City of Scientific Research and Technological Applications (SRTA-City), Alexandria 21934, Egypt; esmailelfakharany@yahoo.co.uk; 5 Center for Microbiology and Phage Therapy, Zewail City of Science and Technology, October Gardens, 6th of October City, Giza 12578, Egypt; 6 Center for X-Ray and Determination of Structure of Matter, Zewail City of Science and Technology, October Gardens, 6th of October City, Giza 12578, Egypt; 7 Key Laboratory of Science and Technology of Eco-Textile, Ministry of Education, College of Chemistry, Chemical Engineering and Biotechnology, Donghua University, Shanghai 201620, China; xmm@dhu.edu.cn

**Keywords:** biopolymer, wound healing, tri-layered, hierarchically structured, self-pumping effect, unidirectional exudates discharge, nanofibrous scaffold

## Abstract

Wound healing has grown to be a significant problem at a global scale. The lack of multifunctionality in most wound dressing-based biopolymers prevents them from meeting all clinical requirements. Therefore, a multifunctional biopolymer-based tri-layered hierarchically nanofibrous scaffold in wound dressing can contribute to skin regeneration. In this study, a multifunctional antibacterial biopolymer-based tri-layered hierarchically nanofibrous scaffold comprising three layers was constructed. The bottom and the top layers contain hydrophilic silk fibroin (SF) and fish skin collagen (COL), respectively, for accelerated healing, interspersed with a middle layer of hydrophobic poly-3-hydroxybutyrate (PHB) containing amoxicillin (AMX) as an antibacterial drug. The advantageous physicochemical properties of the nanofibrous scaffold were estimated by SEM, FTIR, fluid uptake, contact angle, porosity, and mechanical properties. Moreover, the in vitro cytotoxicity and cell healing were assessed by MTT assay and the cell scratching method, respectively, and revealed excellent biocompatibility. The nanofibrous scaffold exhibited significant antimicrobial activity against multiple pathogenic bacteria. Furthermore, the in vivo wound healing and histological studies demonstrated complete wound healing in wounded rats on day 14, along with an increase in the expression level of the transforming growth factor-β1 (TGF-β1) and a decrease in the expression level of interleukin-6 (IL-6). The results revealed that the fabricated nanofibrous scaffold is a potent wound dressing scaffold, and significantly accelerates full-thickness wound healing in a rat model.

## 1. Introduction

The skin tissue serves as a vital barrier against the external environment [1,2]. The skin loses its protective function when exposed to injuries such as wounds or burns, which allows bacteria to enter, causing severe systemic infections [3,4]. Normal wound healing is a complex and dynamic process involving multiple steps, including hemostasis, inflammation, proliferation, and remodeling [5]. The optimal wound dressing materials should act as a barrier between the wound and the external environment to protect the wound site, inhibit the growth of pathogenic micro-organisms, and provide a humid environment around the wound in order to accelerate healing [6,7]. However, a wound site with excessive amounts of wound exudates creates a favorable microenvironment for bacterial growth, which is detrimental to the wound healing process, and thus the removal of the wound exudates is urgently required [8]. Furthermore, it should be fabricated from safe and non-toxic bio-adhesive materials [9,10]. Consequently, developing a wound dressing with unidirectional excessive wound site exudates, draining, and antimicrobial properties is vital for accelerating the wound healing process. In recent years, various strategies involving surface functionalization [11] and in situ polymerization [12] have been developed to fabricate hydrophilic–hydrophobic dual-layer configurations for unidirectional fluid transportation textiles. Nevertheless, the majority of these methods were not cost-effective, lacked robustness, and were difficult to apply [13,14].

Nanotechnology is the study of nano-sized materials and has garnered significant interest due to its numerous applications in the pharmaceutical and biomedical fields [15,16,17,18,19]. The vast majority of skin preparations require frequent reapplication, necessitating the search for the most effective and optimal alternative [20].

Electrospinning is a simple, inexpensive, and widely utilized method for nanofiber manufacturing from a polymer solution with a size ranging from tens of nanometers to micrometers [21]. Nanofibers demonstrate several advantages, such as a small diameter with narrow distribution, a high surface area to volume ratio, excellent mechanical properties, a soft surface with no sharp corners, and increase patient comfort via decreasing the frequency of dressing changes, [17,22,23,24]. In addition to these distinctive characteristics, nanofibers permit skin regeneration, cell respiration, water retention, and effective exudate absorption, making them ideal for wound healing applications [25]. Polymers used in various wound dressing nanofibers can be categorized as either natural or synthetic. The synthesis and modulation of synthetic polymers such as poly(ethylene oxide) (PEO), poly(caprolactone) (PCL), poly(glycolic acid) (PGA), poly(lactic acid) (PLA), and poly(lactic-co-glycolic acid) (PLGA) have considerable flexibility [26,27]. At the same time, natural polymers such as collagen (COL), silk fibroin (SF), and hyaluronic acid (HA) displayed excellent biocompatibility, high safety, biodegradability, low toxicity, and lower immune resistance [28,29]. Due to their poor mechanical strength, high surface tension, and poor solubility in organic solvents, it is challenging to fabricate nanofibers from natural polymers; however, they could be combined with synthetic polymers to produce nanofibers with enhanced mechanical strength [30].

Among these natural polymers, collagen (COL) is a naturally existing animal protein that is abundant in fibrous tissue such as tendons, skin, blood vessels, cartilage, bone, cornea, ligament, and the gut. Fish skin is primarily composed of collagen, mostly type I, characterized by its lower antigenicity and excellent biocompatibility [31].

SF is a natural polymer originating from silk worm cocoons. Its excellent biodegradability, biocompatibility, and convenient mechanical properties make it an excellent material for biomedical areas, including tissue engineering [32,33]. PEO is a biodegradable, biocompatible, chemically stable, and water-soluble polymer that can be spun into nano and microfibers, thus it could be combined with natural polymers to stabilize the electrospinning process [34].

PHB is a hydrophobic, biocompatible, and biodegradable polymer produced by bacteria and has been extensively applied in wound healing and tissue engineering applications [21]. Bacterial infection is considered among the leading causes of delayed wound healing [35]. AMX is a semi-synthetic antibiotic with a broad-spectrum activity against several Gram-positive and Gram-negative micro-organisms [36]. Nonetheless, several reports revealed that systemic administration of antibiotics did not have a significant outcome and was accompanied by several side effects. In addition, the local application of antibiotic powder at the wound site is easily detached from the wound area, causing severe inflammation [36]. The sustained release of antibiotics promoted wound healing by enhancing the nanofiber’s ability to inhibit bacterial growth. 

Due to their varying hydrophilicity and hydrophobicity, unidirectional fluids discharging from electrospun nanofibrous membranes have been extensively used in a variety of biomedical applications [37,38,39]. Utilizing the hydrophilic–hydrophobic gradient structure to generate an additional pressure difference between the hydrophobic region (middle layer) and the hydrophilic region (top layers) could be exploited to achieve unidirectional fluid discharge and self-pumping effects capable of absorbing the excessive wound exudates [40,41,42].

Nevertheless, the majority of electrospun nanofibrous membranes have a bi-layered structure, which limits their applications in preventing reverse osmosis [43]. Therefore, it is anticipated that tri-layered electrospun nanofibrous membranes accelerate the wound healing process due to their improved fluid pumping and the ability of reverse osmosis prevention [44]. Many researchers have developed a tri-layered nanofibrous composite for a wide variety of biomedical applications such as, wound healing [45,46,47], bone tissue regeneration [48], cardiac tissue engineering [49], and tendon rupture repair [50].

To our knowledge, this paper describes, for the first time, the fabrication and design of a tri-layered hierarchically structured nanofibrous scaffold with potent properties based on an electrospinning strategy and sequential layered stacking. The thin layer of electrospun nanofibrous membrane rapidly degrades. The bottom layer consists of a permeable thin layer of electrospun hydrophilic SF, which serves as a source of nutrition for the myo-fibroblasts differentiation and proliferation in the skin extracellular matrix (ECM). The middle layer consists of hydrophobic polymer PHB loaded with antimicrobial drug AMX, respectively, to offer prolonged sustained drug release at the wound site. The hydrophilic polymer COL on the top layer is a component that simulates the ECM to enhance cell growth and absorb the excessive exudates discharged by the unidirectional fluid discharge effect.

The designed tri-layered hierarchically structured nanofibrous scaffold exhibited prolonged drug release, slow in vitro degradation, and a potent antimicrobial effect. The scaffold also exhibited good in vitro biocompatibility. Furthermore, in vivo wound closure, histological and Q-RT-PCR studies on rat models treated with the tri-layered hierarchically structured nanofibrous scaffold demonstrated a complete wound closure, accompanied by increasing the expression level of TGF-β1 and alleviating the expression level of interleukin-6 (IL-6). As a result, we believe that the designed tri-layered hierarchically structured nanofibrous scaffold is a promising candidate for accelerated wound healing applications.

## 2. Materials and Methods

### 2.1. Materials

Raw *Bombyx mori* silk cocoons (*B. mori*) were purchased from the Agricultural Research Institute (Cairo, Egypt). Dialysis tubing cellulose membranes were also purchased (Mwt cut-off 14 Kda, SERVAPOR, HD, Germany). Poly(ethylene oxide) (PEO) (Mwt = 900,000), calcium chloride anhydrous (CaCl_2_), and 2,2,2-trifluoroethanol (TFE) were obtained from Acros-organics (NJ, USA). Poly-(3-hydroxy butyrate) (PHB), amoxicillin sodium (AMX), butyl alcohol anhydrous (CH_3_(CH_2_)_3_OH, glutaraldehyde (GTA), dimethyl sulfoxide (DMSO), dimethylformamide (DMF) and ethanol (EtOH), at HPLC grade, were purchased from Sigma-Aldrich (St Louis, MO, USA). Sodium carbonate anhydrous (Na_2_CO_3_) was procured from Oxford laboratory reagents. Sodium hydroxide (NaOH) was obtained from El-Nasr pharmaceutical chemicals Co. Fetal bovine serum (FBS), Dulbecco’s phosphate-buffered saline (PBS), and Dulbecco’s modified Eagle’s medium (DMEM) were purchased from Gibco-BRL (Grand Island, NY, USA). Acetic acid glacial (CH_3_COOH) was purchased from Oxford laboratory reagents.

### 2.2. Animal and Ethical Approval

Using 21 adult male Wistar rats, the in vivo wound healing rating for the prepared nanofibers was determined (8 weeks old; 180–200 g). The in vivo experimentation complied with the guidelines and protocols and was confirmed by the Research Ethical Committee of Institutional Animal Care and Use Committee at Alexandria University (ALEXU-IACUC) with approval number; AU14-210126-3-3.

### 2.3. Preparation of SF

The SF was prepared according to the previous strategy conducted by Chen, W. et al. [46]. Briefly, raw silk cocoons were degummed in triplicate for 30 min in boiling water containing 0.5% (*w*/*w*) Na_2_CO_3_, followed by washing in distilled water each time. The degummed silk cocoons were dissolved in a CaCl_2_/H_2_O/EtOH solution (molar ratio 1:8:2) at 70 °C under continuous stirring for 1 h. In cellulose dialysis tubes with a 14 Kda cut-off, the solution was dialyzed against distilled water for 3 days at room temperature. Every 5 h, freshly distilled water was changed. The preparation steps ended with filtering, centrifugation, and freeze-drying the solution to obtain a regenerated sf sponge.

### 2.4. Preparation of COL from Tilapia Fish Skin

The fresh tilapia skin was purchased from a local fish market and stored at −20 °C until usage. Fins, fat, and muscle fragments were scraped from the skins before being cut into small sections (0.5 cm^2^ × 0.5 cm^2^) and mixed well. Following Treesin Potaros et al.’s method, acid-solubilized collagen was extracted from the skin of Tilapia fish [51]. Briefly, the fragments were dissolved in 10 volumes of 0.1 M NaOH, and the suspension was agitated with a magnetic stirrer overnight. The skin fragments were re-suspended after decanting in 20 volumes of 0.1 M NaOH solution. The alkaline-insoluble components were filtered through a cloth and repeatedly rinsed with distilled water to achieve a neutral pH. The insoluble parts of the collagen were removed using 10 volumes of 0.5 M acetic acid over the course of three days. Furthermore, the resulting viscous solution was centrifuged at 10,000× *g* for 20 min at 4 °C. The residue was extracted again using 10 volumes of 0.5 M acetic acid for three days, and the extract was then centrifuged. The two extracts’ supernatants were mixed and salted by adding NaCl at a final concentration of 0.9 M. The precipitate was obtained by centrifuging at 10,000× *g* for 20 min after standing overnight. Afterward, it was dissolved in 10 volumes of 0.5 M acetic acid. The solubilization and salting-out processes were carried out three times. The resulting solution was dialyzed against 0.1 M acetic acid in a membrane with a 14,000 kDa cut-off, followed by lyophilization to obtain an acid-solubilized collagen sponge.

### 2.5. Fabrication of a Tri-Layered Nanofibrous Scaffold

The electrospun nanofibrous scaffold was prepared using an electrospinning machine (Nano NC laboratory machine, South Korea Republic, ESR 100) with a grounded aluminum foil-covered drum collector. Briefly, the tri-layered hierarchically structured nanofibrous scaffold was prepared by sequentially layering stacking electrospinning with three different polymeric solutions of SF/PEO, AMX-loaded PHB, and COL/PEO in three different solvents. The bottom layer was made according to the method of Chen et al. [52]; SF/PEO was mixed at a mass ratio of 8:2 and then blended and dissolved in 5 mL distilled water with constant stirring for 24 h at room temperature to obtain the total polymeric concentration of 14% (*w*/*v*). SF/PEO polymeric solution was filled into a 2.5 mL plastic syringe with a blunt-ended needle 7G (ID = 3.81 mm) at a distance of 20 cm from the rotating drum collector at speed = 460 rpm, dispensing rate of 1 mL/h and under applied high voltage of 18–20 Kv. The middle layer was made one according to the method of El-shanshory et al. [21], with some modifications. 

In brief, 0.35 g of PHB was dissolved in 5 mL TFE with constant stirring for 4 h at 50 °C. Subsequently, a PHB with a concentration of 7% (*w*/*v*) was mixed with two concentrations of AMX 5% (*w*/*w*) and 10% (*w*/*w*), depending on the total polymeric solution concentration. PHB and AMX-containing PHB polymeric solution was filled into a 5 mL plastic syringe with a blunt-ended needle 23G (ID = 0.34 mm) at a distance of 20 cm from the rotating drum collector at a speed = 460 rpm, dispensing rate of 1 mL/h and under applied high voltage of 18 Kv. Furthermore, for the top layer, COL/PEO at a weight ratio of 8:2 was blended and dissolved in a 5 mL mixed solvent of glacial acetic acid/DMSO (4.65:0.35) (*v*/*v*) under gentle stirring for 24 h at ambient temperature to obtain a total polymeric concentration of 12% (*w*/*v*). COL/PEO polymeric solution was loaded into a 5 mL plastic syringe with a blunt-ended needle 7G (ID = 3.81 mm) at a distance of 20 cm from the rotating drum collector at speed = 460 rpm, dispensing rate of 1 mL/h and under applied high voltage of 18–20 Kv. For the stabilization and crosslinking of the tri-layered hierarchically structured nanofibrous scaffold against dissolution in fluids, the bottom layer SF/PEO and the top layer COL/PEO were sequentially placed in a sealed desiccator containing 75% EtOH vapor for two days and another sealed desiccator containing 10% (*w*/*w*) GTA vapor for two days, respectively. The stabilized tri-layered hierarchically structured nanofibrous scaffold was dried in a vacuum oven at 40 °C for two days to remove any solvents or crosslinker residuals.

### 2.6. Physicochemical Characterizations

The morphology and tri-layered hierarchically structured nanofibrous scaffold were examined utilizing SEM (JEOL –JSM-6360LA, Tokyo, Japan). Before observation, nanofibrous scaffolds were sputtered with gold. Then, the average diameters of nanofibers were measured using Image analysis software (Image J, National Institute of Health, Bethesda, MD, USA) by randomly selecting 100 nanofibers from the SEM micrographs. FTIR (FT-IR, Shimadzu FTIR-8400 S, Kyoto, Japan) with a wavelength range of 4000–400 cm^−1^ was used to evaluate the composition and chemical structure of the samples.

### 2.7. Swelling, Porosity, and Surface Wettability

The swelling capacity of the samples was determined according to the method conducted by El-shanshory et al. [21]. The initial weight (Wd) of the synthesized nanofibrous sample was recorded, and the sample was placed in phosphate buffer at room temperature. After 24 h, samples were collected, and any excess surface water was gently wiped with filter paper. At this point, the sample’s weight was recorded as (Ww). The following formula was used to calculate the swelling (%).

(1) $$Swelling\left(\%\right)=\left(Ww-Wd/Wd\right)\times 100$$

The porosity of the samples was measured according to the liquid displacement method [53]. A divided cylinder containing a given volume (V1) of absolute ethanol was submerged in a known weight (W) of the sample. When no bubbles were discovered, the resulting volume was then reported (V2). Finally, the absolute ethanol volume remaining after the sample was removed from the absolute ethanol was determined (V3). The porosity of the sample was determined according to the following equation:

(2) $$Porosity\left(\%\right)=\left((V1-V3\right)/\left(V2-V3)\right)\times 100$$

The surface wettability of the nanofibrous scaffold samples was evaluated using a contact angle meter, model VCA 2500 XE, with a CCD camera and software (AST Products, Billerica, MA, USA). After 0.03 mL of deionized water was dropped onto the surface of the nanofibrous scaffolds for 1 s, images were captured using the connected camera.

### 2.8. Mechanical Properties Evaluation

The tensile strength of nanofibrous scaffolds at room temperature was measured using a universal testing machine (Shimadzu UTM, Kyoto, Japan). Crossheads were moved at a constant rate of 5 mm/min at room temperature until samples rupture (n = 5), while tensile strength was measured automatically. The elongation at rupture and tensile strength were calculated.

### 2.9. In Vitro Antimicrobial Activity

Antimicrobial agents can kill or inhibit bacterial growth. Several techniques, including agar dilution, disc -diffusion, and well diffusion, have been successfully applied to evaluate and screen antimicrobial activity [54]. In this study, the antimicrobial activities of the fabricated nanofibers (SF/PEO/PHB/COL/PEO, SF/PEO/AMX/PHB/COL/PEO) were evaluated against Staphylococcus epidermidis ATCC 12228, Escherichia coli ATCC 25922, Staphylococcus aureus ATCC 29213, and Enterococcus faecalis ATCC 29212, according to previously reported methods [55]. Briefly, the previously refreshed bacteria suspensions were diluted with sterile 1% LB broth medium up to 100 fold, then 100 μL of the diluted bacterial suspension was incubated with 10 mL of sterile 1% LB medium containing 0.030 g of tested sample (nanofibers) while shaking for 24 h at 37 °C. Evaluation of the absorbance of the culture medium at 600 nm with visible spectroscopy revealed the percentage of bacterial growth inhibition. The percentage of bacterial inhibition was calculated according to the following equation.

(3) $$\%\;inhibition\;\frac{A-B}{A}\;\times \;100$$

where A and B are the absorbances of bacterial culture in the absence and the presence of tested nanofiber, respectively.

### 2.10. Drug Release Assessment

To evaluate the in vitro drug release of nanofibrous scaffolds containing AMX, a calibration curve was generated by measuring the absorbance values of progressively diluted AMX concentrations at 273 nm. In brief, 3 mL of PBS buffer was added to two cassettes containing 10 mg of two concentrations of AMX-loaded nanofibrous scaffold. The cassettes were then placed in a shaker incubator set to 100 rpm at 37 °C. The buffer was removed, and the same amount of new PBS buffer was added at a predetermined time. The UV spectrophotometer-double beam (T80+, PG Instruments Ltd., England, UK) was utilized for absorbance detection. Calculations were performed based on the AMX concentration in the buffer, the AMX release percentage, and the cumulative release curve.

### 2.11. Cytotoxicity Assay of Nanofibrous Scaffolds

Following treatment, the effect of nanofibrous scaffolds on normal human cells of the HSF (Primary skin fibroblasts) and HFB-4 (melanocytes) cell lines was evaluated using the MTT cell viability assay. In 24-well sterile flat-bottom tissue culture plates, HSF and HFB-4 cells (1.0 × 10^3^ each) were cultivated for 24 h in a CO_2_ incubator. HSF and HFB-4 cells were maintained in full DMEM media supplemented with 10% FBS. In triplicates, the discs from each manufactured NF were cultured in the monolayer cells for two and four days at weights of 0.5, 1.0, 2.0, and 4.0 mg/mL. After three rounds of washing with a new medium, cells were treated with a 0.5 mg/mL MTT solution to remove dead cells and debris before being cultured for approximately 2–3 h in 5% CO_2_. The formed formazan crystals were dissolved in DMSO, and the optical density of each well was measured at 590 nm using an ELISA reader and a microplate. Without including the prepared NFs, the relative cell viability (%) in comparison to reference cells was calculated using the formula (X) test/(Y) reference 100%.

### 2.12. Cell Scratching Assay of Nanofibrous Scaffolds

The effect of cell healing on the manufactured nanofibrous scaffold was determined using the cell scratching method. Briefly, sterile 24-well cell culture plate (HFB-4, 1.0 × 10^5^) cells were cultivated and then incubated in 5% CO_2_ until the cell monolayers reached about 90% confluence. The cells were washed with fresh medium after being scratched by a sterile micro-tip on the monolayers. After adding different discs of the nanofibrous scaffolds to each well individually, they were incubated in 5% CO_2_ for 24 and 48 h to allow for cell migration in the medium. Scratching healing and cell migration were then visualized and recorded using a phase contrast microscope. Each experiment was carried out three times, and the results were compared to untreated scratched cells.

### 2.13. In Vivo Wound Healing

The in vivo wound healing rating for the nanofibrous scaffolds was executed using 21 adult male Wistar rats (8 weeks old; 180–200 g). The in vivo experimentation followed the guidelines and protocols and was approved by the Research Ethical Committee of the Institutional Animal Care and Use Committee at Alexandria University (ALEXU-IACUC) with approval number; AU14-210126-3-3. The animals were divided into three groups, with seven animals in each one. Group one received sterile gauze, group two received SF/PEO/PHB/COL/PEO (blank), and group three received SF/PEO/AMX/PHB/COL/PEO. All groups were covered with plaster to secure the wound dressing at the wound place. The rats were housed in separate stainless-steel cages, supplied with a standard laboratory diet and mineral water ad libitum, and kept under planned environmental conditions (50–60% humidity and 12 h light/dark cycle at 25 ± 2 °C) for 7 days prior to the experiment to allow for adaptation. The surgical operation was performed as previously reported [2,4,56,57]. The rats were anesthetized with intramuscular injection of 10% Ketamine Hydrochloride “(Dopalen^®^-Sespo Indústria e Comércio Ltda, Vetbrands Saúde Animal Division, Paulínia, Brazil, 0.1 mL/100 g body weight) and 2%xylazine hydrochloride (Calmium^®^-Agener União, União Química, Embu-Guaçu, SP, Brazil, 0.1 mL/100 g body weight)”, the back hair was shaved using an electric animal shaver, followed by sterilization of the skin using ethanol solution (70%) and chlorhexidine. Then, using sterile surgical scissors, a surgical scalpel, and forceps, a 1.5 cm diameter, a full-thickness circular excisional wound was created in the center of the hairless skin. The dressed nanofibrous scaffolds and sterile gauze were substituted for new ones every 3 days for 14 days. The wound areas were calculated with the help of a digital caliper. The macroscopic photos of the wounds were captured to evaluate the % contraction of the wound area using a digital camera on days 0, 3, 7,10, and 14 immediately after the surgical operation. The percentage of wound area reduction was estimated according to the following equation.

Wound area reduction (%) = [1 − (Wound area at the given day/Wound area at the day 0)] × 100.(4)

For histological examination, the skins from the wound sites were removed before scarification on the 7 and 14 days and then fixed in 10% formalin prior to slide preparation. Subsequently, the skin slides were stained with both hematoxylin and eosin (H&E) and Masson’s trichrome (MTS) and then examined under an optical microscope for epithelialization, keratinization, and collagen deposition [17,53]. Finally, a section of the skin tissue was preserved at −80 °C for further molecular analysis.

### 2.14. Quantitative Real-Time Polymerase Chain Reaction (RT PCR) Gene Expression Assay for Interleukin-6 and TGF-β1

The total tissue RNA was isolated from the wounded skin samples collected on day 14 using the Easy-spinTM Total RNA extraction kit (cat. No. 17221, South Korea, iNtRON Biotechnology) following the manufacturer’s instructions. The purity and the concentration of the extracted total RNA were evaluated utilizing a NanoDrop™ UV–vis spectrophotometer. Using the cDNA synthesis kit TOPscriptTM (cat. No. EZ005S, Daejeon, South Korea, Enzynomics, Inc.), the reverse-transcription step for converting RNA to complementary DNA (cDNA) was performed. The RT-PCR amplification reactions were performed using SYBR green qPCR Master Mix (Thermo Fisher Scientific, Inc., Waltham, MA, USA, cat. No. K0251) utilizing real-time PCR system, Applied Biosystems with 2.5 μL of cDNA and 1.5 μL)of each primer in a 25 μL reaction mixture final volume. The expression level of the house-keeping gene Beta-actin (β-actin) was applied as the internal control for the amplified samples. The sequences for the primers used for the amplification of (cDNA) were as follows: 5-TTTCTCTCCGCAAGAGACTTCC-3 (forward) and 5-TGTGGGTGGTATCCTCTGTGA-3 (reverse) for IL-6; 5-TGACATGAACCGACCCTTCC-3 (forward) and 5-TGTGGAGCTGAAGCAGTAGT-3 (reverse) for TGF-β1 and 5-AGATCAAGATCATTGCTCCTCCT-3 (forward) and 5-ACGCAGCTCAGTAACAGTCC-3 (reverse) for β-actin. The alterations in the level of gene expression were estimated using a delta delta comparative Ct (2-ΔΔCt) analysis technique. For the examined genes, the amplification technique consisted of one cycle of initial denaturation at 95 °C for 10 min, followed by 40 cycles of 15 s at 95 °C, 30 s at 57 °C and 30 s at 72 °C. Melting curve analyses were performed for all amplifications to ensure a single product was generated from each reaction [58].

### 2.15. Statistical Analysis

The collected data were statistically analyzed using costate software. One-way ANOVA was performed, followed by the LSD test for multiple comparisons. Data were expressed using the mean and standard deviation M ± SD. *p* ≥ 0.05 was regarded as statistically significant across all analyses.

## 3. Results and Discussion

### 3.1. Preparation and Physicochemical Characterization

**Figure 1:**
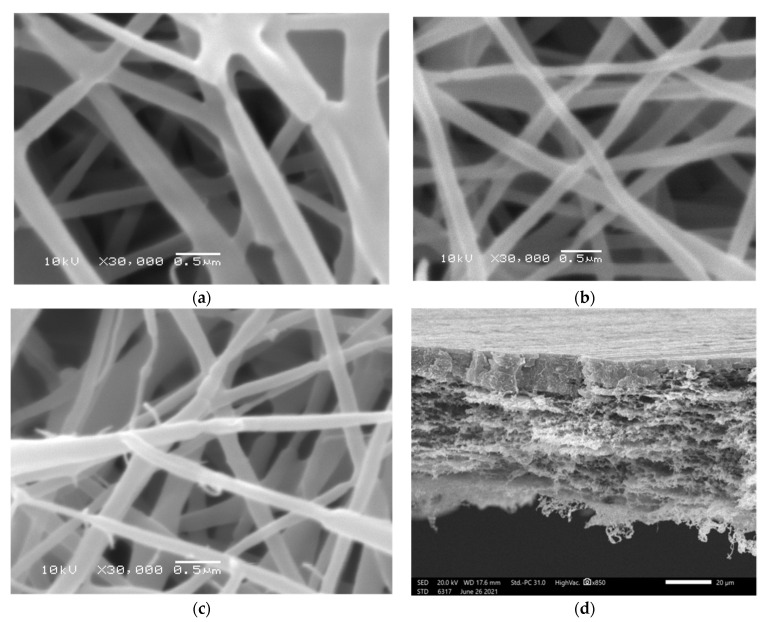
Nanofibrous scaffold morphology. SEM images of SF/PEO (**a**), PHB (**b**), and COL/PEO (**c**), and a cross-sectional micrograph of a tri-layered hierarchically structured nanofibrous scaffold (**d**).

The morphological appearance and tri-layered hierarchical structures of the as-prepared nanofibrous scaffolds (Figure 1) were evaluated under magnification, and the distribution of their average diameters is depicted in supplementary data (Figures S1–S3). Smooth surfaces, beadless, and no spindle on a string behavior were detected. Moreover, no AMX accumulations were detected on the surface of nanofibrous scaffolds. According to these results, the size distribution and average diameters are 250 ± 82 nm, 261 ± 49 nm, as well as 266 ± 55 nm for SF/PEO, PHB, and COL/PEO, respectively.

**Figure 2:**
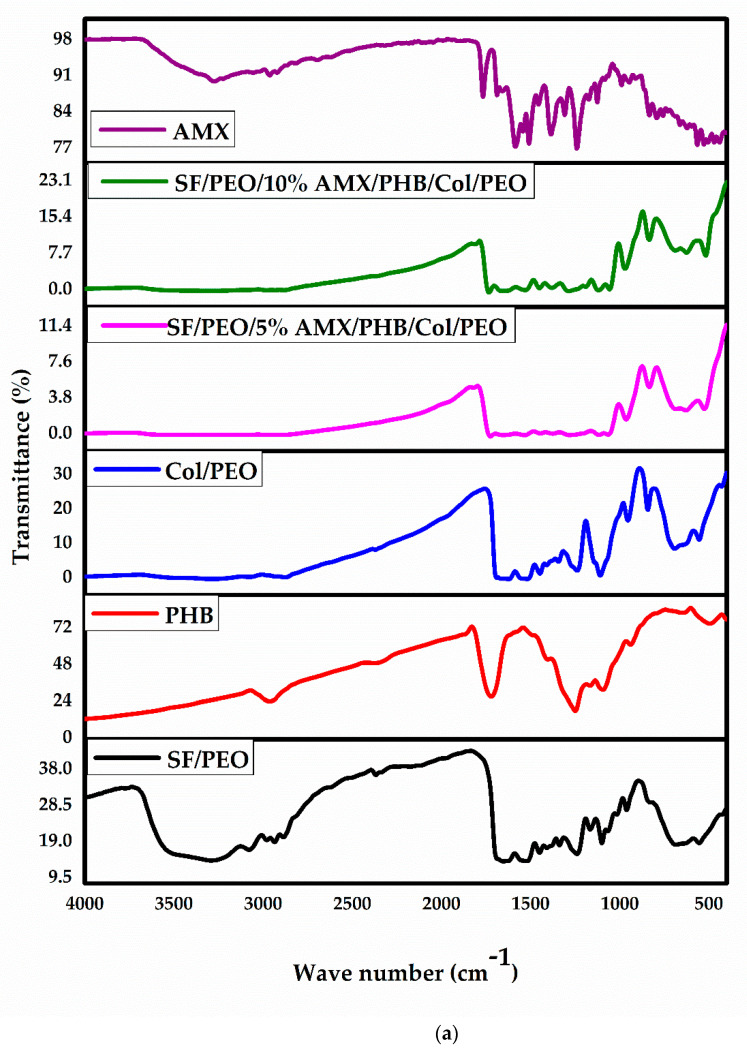

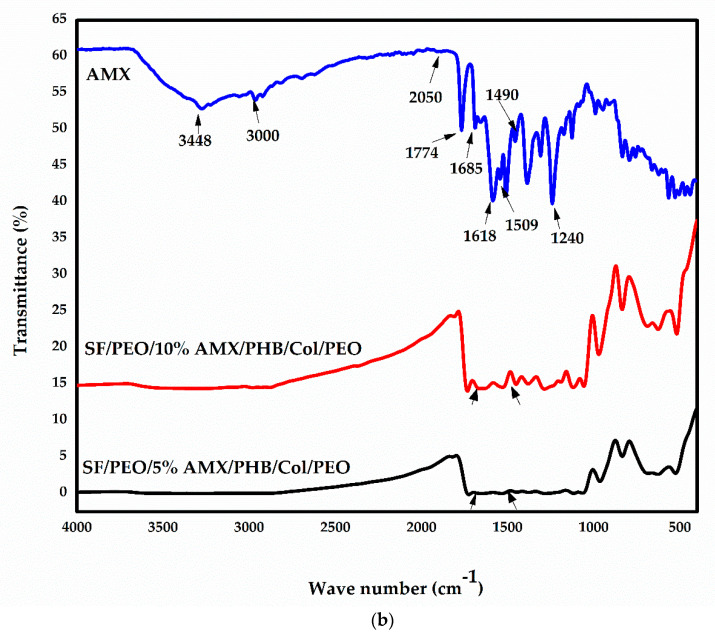
(**a**) FT-IR spectra of SF/PEO, PHB, COL/PEO, SF/PEO/5%AMX/PHB/COL/PEO, SF/PEO/5%AMX/PHB/COL/PEO and AMX. (**b**) FT-IR spectra of SF/PEO/5%AMX/PHB/COL/PEO, SF/PEO/5%AMX/PHB/COL/PEO, and AMX.

FT-IR was evaluated to detect the characteristic peaks and functional groups for the ingredients of the as-prepared nanofibrous scaffolds SF, PHB, and COL. The basic characteristic peaks of SF/PEO are explained by the appearance of amide I, C=O stretching bands and were assisted by the presence of amide II and III at 1535 and 1238 cm^−1,^ respectively. Furthermore, the random coil of amide I appeared at 1654 cm^−1^, corresponding to the vibration band. PHB exhibited infrared absorption peaks at 1262 and 1725 cm^−1^, corresponding to –CH and C=O, respectively, present in the ester group in the molecular chain. Additionally, sharp peaks at 1034 and 1097 cm^−1^ represent C–O stretching. Furthermore, absorption peaks at 2926 and 2963 are attributed to C–H stretching vibrations of the methyl and methylene groups [59]. AMX relevant major peaks are present at 1490, 1509–1520, 1685–1692, 2050, 3000, 3175, 3366, and 3448–3458 cm^−1^ corresponding to N–H, C=C benzene ring stretching, C=O stretching, amide I, C–C and C–N stretching, C–H benzene ring stretching and amide N–H and phenol O–H stretching, respectively [60,61]. Collagen-derived tilapia fish skin showed characteristic peaks at 3292–3315 cm^−1^ corresponding to peptide N–H groups. There are COL amide I band at 1656 cm^−1^, amide II at 1538–1548 cm^−1^, and amide III at 1232–1238 cm^−1^. Therefore, these results confirmed that the extracted COL is collagen type I which are in concordance with the previously reported results by Elbaily et al. [62]. The successful loading of AMX within the nanofibrous composite can be confirmed by the appearance of the peaks in AMX powder at 1509 cm^−1^ and 1685 cm^−1^, which are assigned to amide I and Amide II bond of AMX, respectively [63]. Moreover, the peaks at 1618 cm^−1^, 1774 cm^−1^ and 3448 cm^−1^ are present due to the absorption band of benzene ring, the vibration of carboxylic group and the stretching vibration of hydroxyl and amino group in the AMX structure [64,65]. Additionally, the appearance of the AMX peak at 1509 cm^−1^ in both SF/PEO/5%AMX/PHB/COL/PEO and SF/PEO/10%AMX/PHB/COL/PEO confirms the presence of AMX in both composites due to some weak van der Waals interactions between AMX and nanofibrous composite. However, the detection of other AMX signals was difficult perhaps due to some overlapping between vibration bands of AMX and the nanofibrous composites [66]. The results obtained indicated the successful incorporation of AMX into the nanofibrous scaffold. FT-IR graphs are demonstrated and plotted in Figure 2.

The nanofibrous scaffold’s ability to manage wound exudates and drug delivery depends on its ability to absorb fluids. Fluids uptake is affected by the hydrophilic nature of the applied materials and their porosity. The swelling ability of the material affects its weight, which decreases due to erosion during prolonged exposure to fluids, whereas weight gain is due to fluid uptake during short exposure to fluids. The weight change obtained after 72 h of immersion in PBS pH 7.4 at room temperature with partial weight loss in the first 24 h was 25%, 5%, and 65% for SF/PEO, PHB, and COL/PEO, respectively. Furthermore, All nanofibrous scaffolds maintained their structural stability for at least 48 h. These results demonstrate the potency of the nanofibrous scaffold for various biomedical applications. Additionally, the porosity % of the nanofibrous scaffolds is a key factor affecting their biomedical applications. The porosity % for SF/PEO, PHB, SF/PEO/PHB/COL/PEO (blank), and COL/PEO are 75%, 80%, 85.71%, and 82.2%, respectively. These results demonstrate the suitability of the materials for use in biomedical applications and the capacity of the nanofibrous scaffold to facilitate the self-pumping effect and fluid movement from the interior to the exterior surface of the matrix.

**Figure 3:**
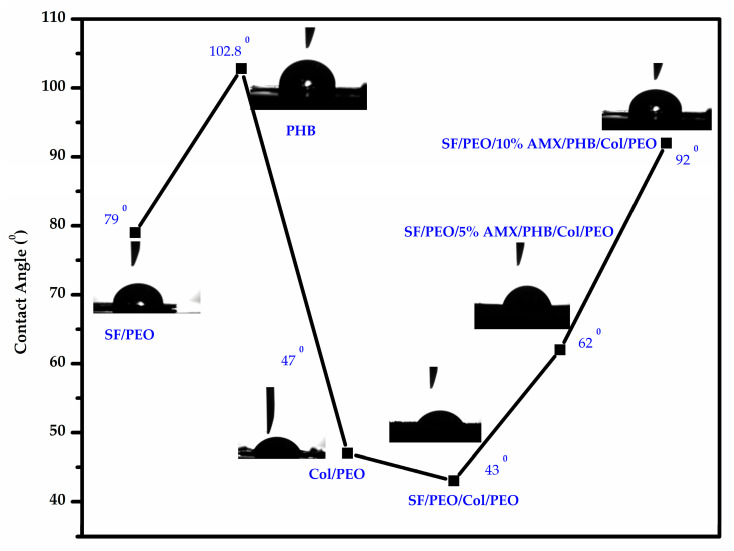
Photographic demonstration of contact angle values for SF/PEO, PHB, COL/PEO, SF/PEO/COL/PEO, 5%, and 10% AMX incorporated nanofibrous scaffolds.

The hydrophilic behavior of nanofibrous scaffold significantly enhances cell differentiation and adhesion. In general, the water contact angle of the SF/PEO is 79.2°, while that for PHB is 102.8°. Moreover, the water contact angles for the COL/PEO, SF/PEO/COL/PEO, 5% AMX-loaded nanofibrous scaffold, and 10% AMX-loaded nanofibrous scaffold were 47°, 43°, 62°, and 92°, respectively. The photographs of water contact angles of the different nanofibrous scaffolds are shown in Figure 3. The obtained results indicate the hydrophobic nature of PHB, the moderate hydrophilicity of SF, COL, SF/PEO/COL/PEO, and the change in behavior of the 10% AMX-incorporated nanofibrous scaffold towards hydrophobicity.

Materials used in biomedical applications should have suitable mechanical properties to serve well as a scaffold. The results summarized in Table 1 indicated that the SF/PEO has a tensile strength of 9.1911 N/mm^2^ and the value of elongation at break was 2.70500%, whereas the tensile strength of COL/PEO is 27.777 N/mm^2^, and the value of elongation at break was 1.1100%, Moreover, adding PHB and COL/PEO to SF/PEO layers exhibited a tensile strength value for SF/PEO/PHB/COL/PEO scaffold to be 4.1666 N/mm^2^ and the value of elongation at break decreased to be 0.6000%. The incorporation of AMX (5%) in SF/PEO/PHB/COL/PEO increased the values of tensile strength and elongation at break, respectively, from 4.1666 N/mm^2^ to 35.1732 N/mm^2^ and 0.6000% to 1.84500%. The addition of AMX(10%) in SF/PEO/PHB/COL/PEO increased the tensile strength and elongation at break values, respectively, from 4.1666 N/mm^2^ to 86.137 N/mm^2^ and 0.6000% to 2.50250% [67,68,69].

### 3.2. In Vitro Antimicrobial Activity

In several cases, wound infection may lead to severe problems and complications associated with delayed healing as well as mortality. Consequently, it is necessary for the newly developed wound dressing to estimate its ability to inhibit the growth of pathogenic bacteria. The antibacterial activity was assessed regarding the % growth inhibition of *Staphylococcus epidermidis* ATCC 12228, *Escherichia coli* ATCC 25922, *Staphylococcus aureus* ATCC 29213, and *Enterococcus faecalis* ATCC 29212bacteria after one day of contact with the nanofiber. These bacteria are prevalent in wound discharges, especially in post-operative patients. SF/PEO/PHB/COL/PEO (blank) did not inhibit bacterial growth because it lacked antimicrobial ingredients. Conversely, SF/PEO/5%AMX/PHB/COL/PEO demonstrated highly significant antimicrobial activity against *Staphylococcus epidermidis*, *Escherichia coli*, *Staphylococcus aureus*, and *Enterococcus faecalis*, with the calculated reduction in the number of CFU of these bacterial cells reaching maximum of 83, 76.5, 83, and 89.5%, respectively.

### 3.3. In Vitro Drug Release and In Vitro Weight Loss

**Figure 4:**
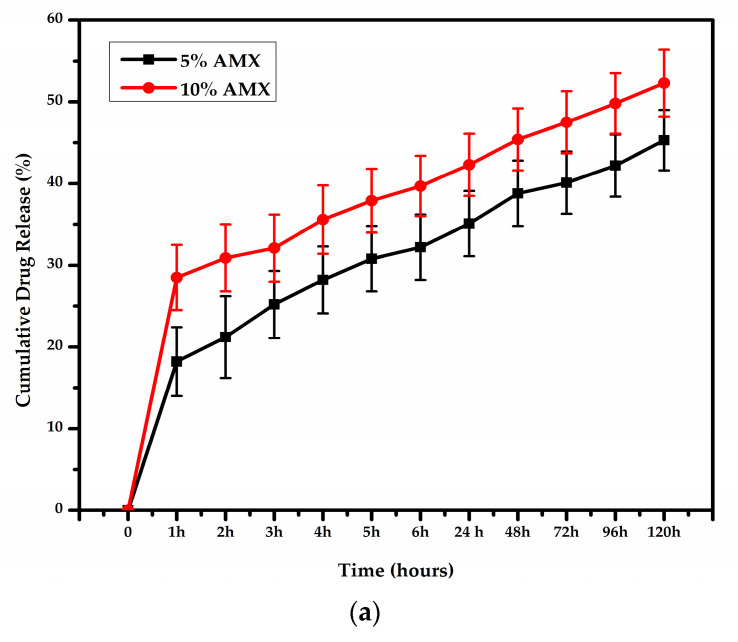

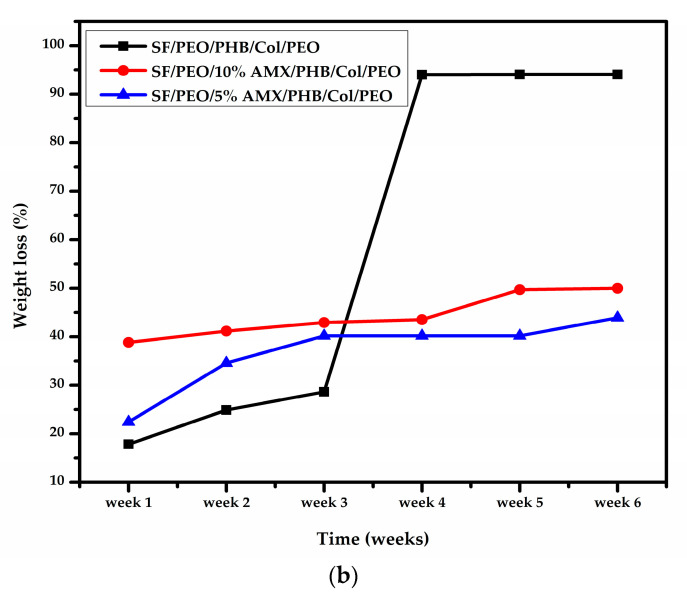
Photographic illustration of (**a**) in vitro release profiles of AMX from SF/PEO/5%AMX/PHB/COL/PEO and SF/PEO/10%AMX/PHB/COL/PEO and (**b**) in vitro weigh loss of SF/PEO/PHB/COL/PEO, SF/PEO/5%AMX/PHB/COL/PEO, and SF/PEO/10%AMX/PHB/COL/PEO.

Figure 4 summarizes the in vitro cumulative release profiles of nanofibrous scaffolds incorporating 5% and 10% AMX. In addition, the AMX concentration in the release media was plotted versus time to determine the amount of drug released in vitro from nanofibrous scaffolds. The release of the drug was detected using a UV spectrophotometer and lasted up to 120 h.

Additionally, the degradation rate of the nanofibrous scaffolds incubated in PBS solution at 37 °C was conducted for up to 6 weeks, as shown in Figure 4b. For the nanofibrous scaffold containing 5% AMX, the weight loss percentage at week 1 was 22.4%, whereas at week 6, it was 43.9%. In contrast, the percentage of weight loss at week 1 and week 6 for the nanofibrous scaffold containing 10% AMX was 38.8% and 50%, respectively.

Previous studies reported that AMX is more soluble in acidic media resulting in a decrease in the swelling of the polymeric matrix with increasing AMX concentration [70]. Moreover, electrospinning process allows hydrophobic components of AMX to face the surface of polymers due to the rapid solvent evaporation rate and this results in polymer degradation retardment in the presence of AMX. These results are in agreement with the results reported previously by Mollo et al. [71]. Based on the obtained results, we hypothesize that a nanofibrous scaffold containing 5% AMX can serve as an effective scaffold against bacterial invasion for wound healing applications.

### 3.4. In Vitro Cytotoxicity and Cell Scratching Assay

**Figure 5:**
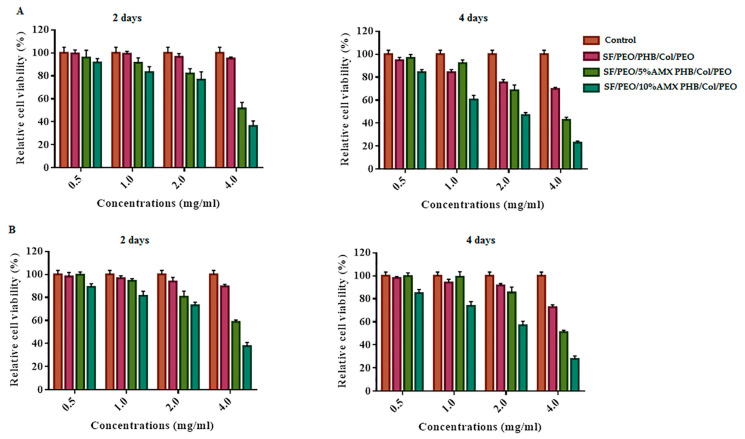
In vitro cell viability assessment: cytotoxic effect test of the prepared NFs by MTT assay on both HFB-4 (**A**) and HSF; (**B**) human normal skin cell lines after 2 and 4 days of incubation.

**Figure 6:**
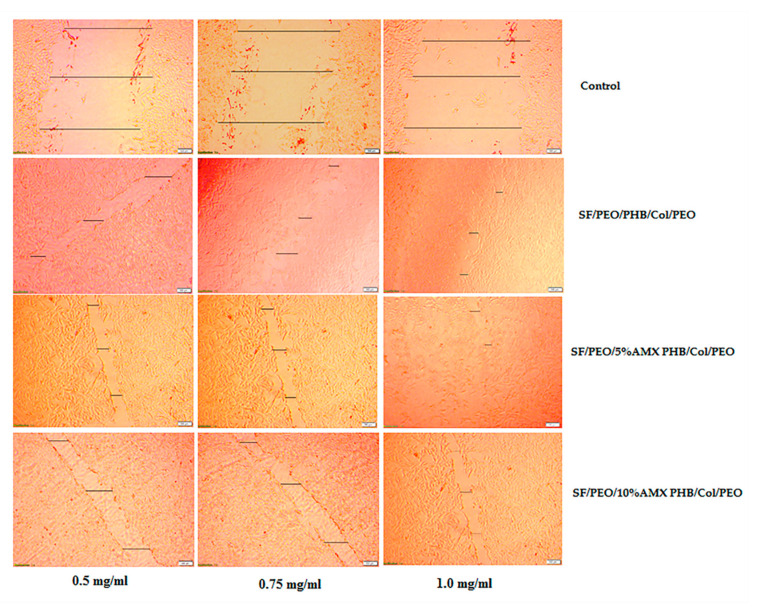
Typical photomicrographs of cell scratching of HFB-4 melanocytes at 48 h (scale bar is 500 µm). The black bars indicate the distances of the wound closure. All experiments were performed three times.

Cell viability and the effect of cell healing by the cell scratching method, as depicted in Figure 5 and Figure 6, are binding assays for scaffolds used in wound healing and other biomedical applications. According to Figure 5, the cell viability of all tested nanofibrous scaffolds after two and four days of incubation ranged between 50 and 80%, indicating that these nanofibrous scaffolds have some cytotoxic effects on cells exposed to them for four consecutive days. Consequently, based on the obtained results, it can be concluded that blank nanofibrous scaffolds under study are cytocompatible, allowing more than 80% cell viability, while the nanofibrous scaffold containing 5% and 10% AMX were found to be almost 35–50% cell viability.

Moreover, from the results obtained from the cell scratching assay, the blank and nanofibrous scaffold incorporated with 5%AMX at different cell concentrations performed well for wound healing. In contrast, the nanofibrous scaffold incorporated with 10%AMX showed relatively lower wound healing and some toxic effect on the cells. Based on these findings, the tri-layered hierarchically structured nanofibrous scaffold is recommended for wound healing applications. Furthermore, The viability of PIEC cultured on pure SF, SF/P(LLA-CL) and SF/HBC nanofibrous scaffolds were good and beneficial to cell growth in comparison with coverslips as reported by Zhang et al. [72,73]. Further, the previous studies conducted by Tian Zhou et al. have demonstrated that the fish collagen/BG nanofibers induced proliferation on HaCaTs, indicating that fish collagen nanofibers could effectively promote wound healing [74]. Moreover, the cell viability of HPDLCs cultured was enhanced upon seeding on the Col/BG/CS membrane for periodontal tissue regeneration as reported by Zhou et al. [75]. Additionally, it has been reported that the biocompatibility results of the PHB/gelatin nanofibers against NIH-3T3 fibroblast cell lines were found to be non-toxic and aid in greater cell viability [76]. Additionally, it has been reported that the combination of PHB with SF supported the cell attachment and proliferation of L929 and HaCaT cell lines [77].

### 3.5. In Vivo Wound Healing

**Figure 7:**
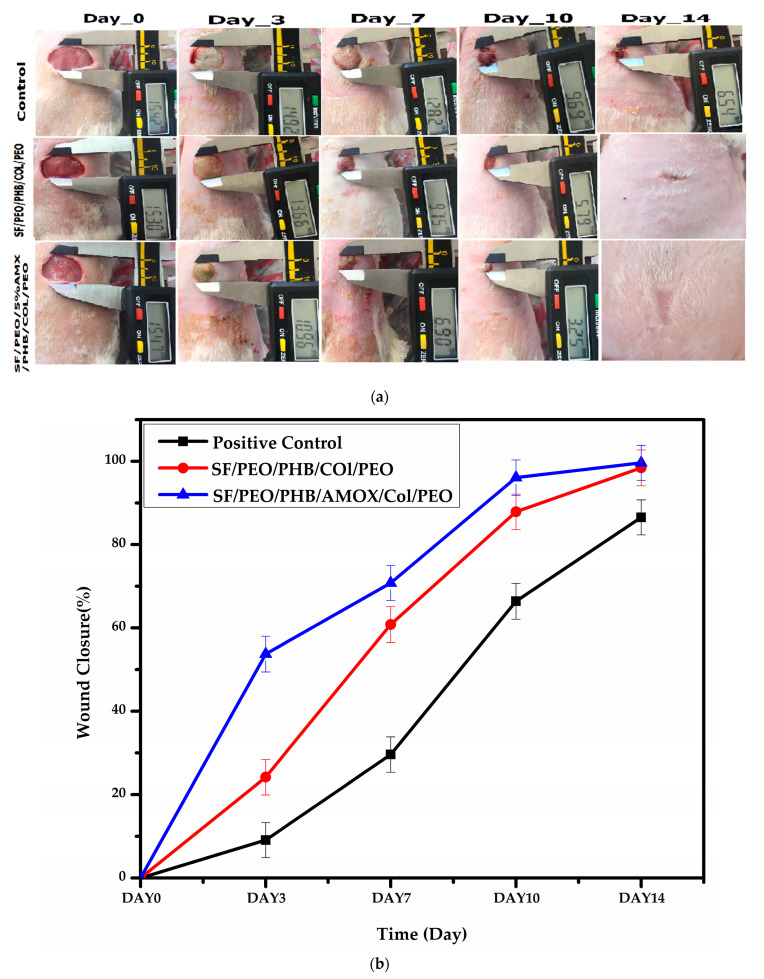
(**a**): Photographic images of in vivo full-thickness excision wounds after treatment with sterile gauze, collagen/silk fibroin NFs, and collagen/AMX/silk fibroin NFs for 14 days. (**b**) The graph illustrates (%) wound contraction during 14 days.

The effect of different treatment groups on in vivo wound healing efficiency is illustrated in Figure 7a and Table 1. Topical delivery of the antibiotics leads to bulk collection of higher drug doses at the target site, thus reducing side effects such as systemic toxicity associated with high drug doses and bacterial resistance. Therefore, the optimal wound dressing material should contain antimicrobial agents with a sustained release behavior in order to accelerate wound healing [78]. Additionally, in contrast to free antibiotics that cannot arrive and spread evenly over infected areas, antibiotic-loaded nano-carriers are characterized by their higher penetration efficiency and equal distribution around the infected spots [79]. In this regard, AMX, a broad-spectrum antimicrobial compound, was incorporated into the SF/PEO/PHB/COL/PEO nanofibrous scaffold to boost wound repair. In comparison to the sterile gauze group, the SF/PEO/PHB/COL/PEO (blank)- and 5% AMX incorporated SF/PEO/5%AMX/PHB/COL/PEO nanofibrous scaffold-treated groups displayed significant progress in wound healing associated with normal tissue, with no exudates at all healing stages.

In addition, the incorporation of AMX into the SF/PEO/PHB/COL/PEO nanofibrous scaffold accelerated the wound healing process. The wounds treated with the SF/PEO/5%AMX/PHB/COL/PEO nanofibrous scaffold were partially healed after 7 days. The % contraction of the wound area was studied as shown in Table 2 and Figure 7b. It was found that after 3 days from treatment, the percentage of wound closure was 9.09%, 24.18%, and 53.73% for the sterile gauze group, the SF/PEO/PHB/COL/PEO nanofibrous scaffold, and the SF/PEO/5%AMX/PHB/COL/PEO nanofibrous scaffold, respectively. Interestingly, at day 14 after treatment, both the SF/PEO/5%AMX/PHB/COL/PEO nanofibrous scaffold and the SF/PEO/PHB/COL/PEO nanofibrous scaffold showed 99.63% and 98.40% wound closure, respectively. Conversely, the sterile gauze group showed a wound closure of 86.54%. The previous results showed that the wound closure was slower in the sterile gauze group than in the nanofiber-treated groups.

**Figure 8:**
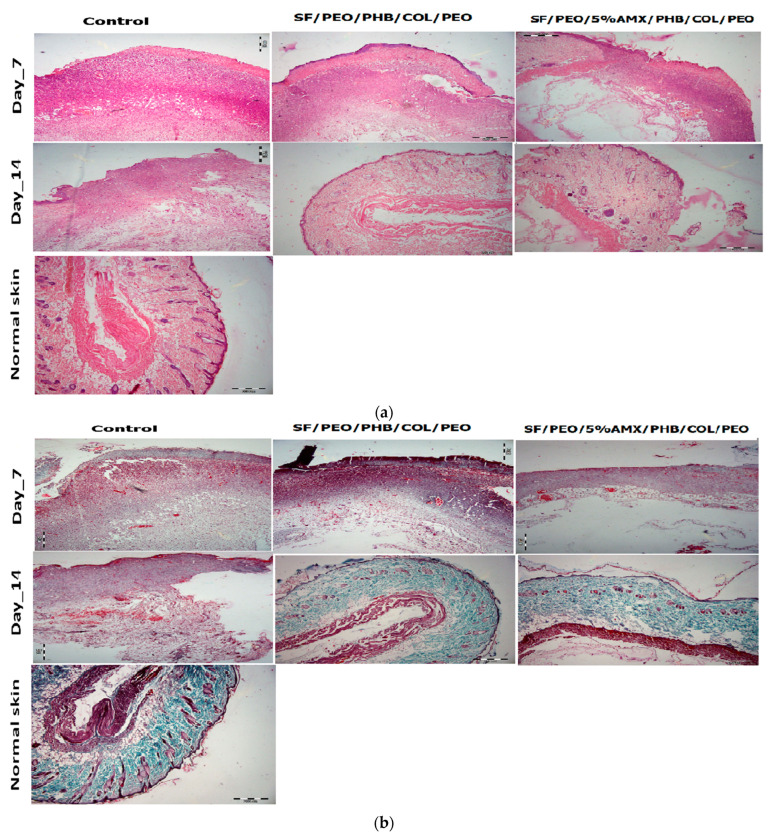
(**a**) Photographic illustration of histological analysis of treated wounds on days 7 and 14 using H&E stain. (**b**) Histopathological evaluation of treated wounds using MTS on days 7 and 14 (original magnification = 100).

The histopathological examination of the skin sections from different treated groups was performed to estimate the degree of vascularization, inflammation, and skin regeneration. During the early stage of wound healing, the lower inflammation level can promote factors responsible for wound healing. On the other hand, severe inflammation in wound sites hindered tissue repair. Figure 8a,b showed the histopathological features of H&E and MTS stained skin sections of NF-treated wounds compared with untreated control and normal skin on days 7 and 14 post-wounding, respectively. As depicted in Figure 8a, the normal skin image presented the typical structural elements of healthy skin, such as intact epidermis, dermis (connective tissue layer), muscles, sebaceous glands (SG), blood vessels, and hair follicles. It is evident that the healing of the skin section treated with the SF/PEO/PHB/COL/PEO nanofibrous scaffold and the SF/PEO/5%AMX/PHB/COL/PEO nanofibrous scaffold was higher compared to the sterile gauze group. Furthermore, the nanofibers loaded with AMX showed considerable healing over the early stages of post-operation. On day 7, the skin section from groups treated with sterile gauze showed ulcers, inflammatory cells, no SGs, and intact hair follicles. On the contrary, the SF/PEO/PHB/COL/PEO and SF/PEO/5%AMX/PHB/COL/PEO nanofibrous scaffold-treated group begins forming a thin coat of neo-epithelium associated with mild inflammatory cells, mild new vessel, and fibroblasts. On day 14, the wound section from the sterile-gauze-treated group showed a small ulcer, scab formation with a smaller number of fibroblasts and vasculature, and ahigh number of inflammatory cells with minimal re-epithelialization and the absence of both regenerated SGs, hair follicles, and other adnexa in the renovated dermis. The wound site also showed loosely arranged connective tissue bundles in the dermis. Conversely, the wound sections of SF/PEO/PHB/COL/PEO and SF/PEO/5%AMX/PHB/COL/PEO nanofibrous scaffold-treated group showed relatively normal skin appearance loaded with connective tissues (CT) and enveloped by a new dermal layer. The tissues contained a fully developed epidermis (2–3 layers) and dermis (completely organized connective tissue layer). The wound section also displayed the most basic skin structures, such as hair follicles, SG, and blood vessels. The degree of collagen formation and deposition from the wound section were estimated among various groups by using Masson’s trichrome staining (MTS) Figure 8b. Sterile-gauze-treated groups showed loose collagenous fiber deposition at the lesion site on both day 7 and day 14. In contrast, the highest collagen fiber deposition was observed in both SF/PEO/PHB/COL/PEO and SF/PEO/5%AMX/PHB/COL/PEO nanofibrous scaffold-treated groups characterized by mild collagen deposition on day 7, which became denser on day 14 and resembled the pattern of collagen deposition in the normal skin (basketweave), which confers both flexibility and pliability [22]. The photographic images and histopathological examination results suggested that the SF/PEO/5%AMX/PHB/COL/PEO nanofibrous scaffolds accelerate wound healing by promoting complete re-epithelialization, collagen deposition, and arrangement. The efficient in vivo wound healing performance was previously reported for collagen and zein nanofibrous membranes loaded with berberine [80]. Another study observed an accelerated in vivo wound healing, re-epithelization, and collagen deposition for collagen nanofibers loaded with AgNPs due to their intrinsic antibacterial activity [81]. The hybrid between collagen and silk fibroin might improve both the physical and biological characteristics of the scaffold for both tissue engineering and biomedical applications. This result may be due to the hybrid’s resemblance to the ECM skin, which promotes cell adhesion and proliferation [82].

### 3.6. Gene Expression of IL-6 and TGF-β1

**Figure 9:**
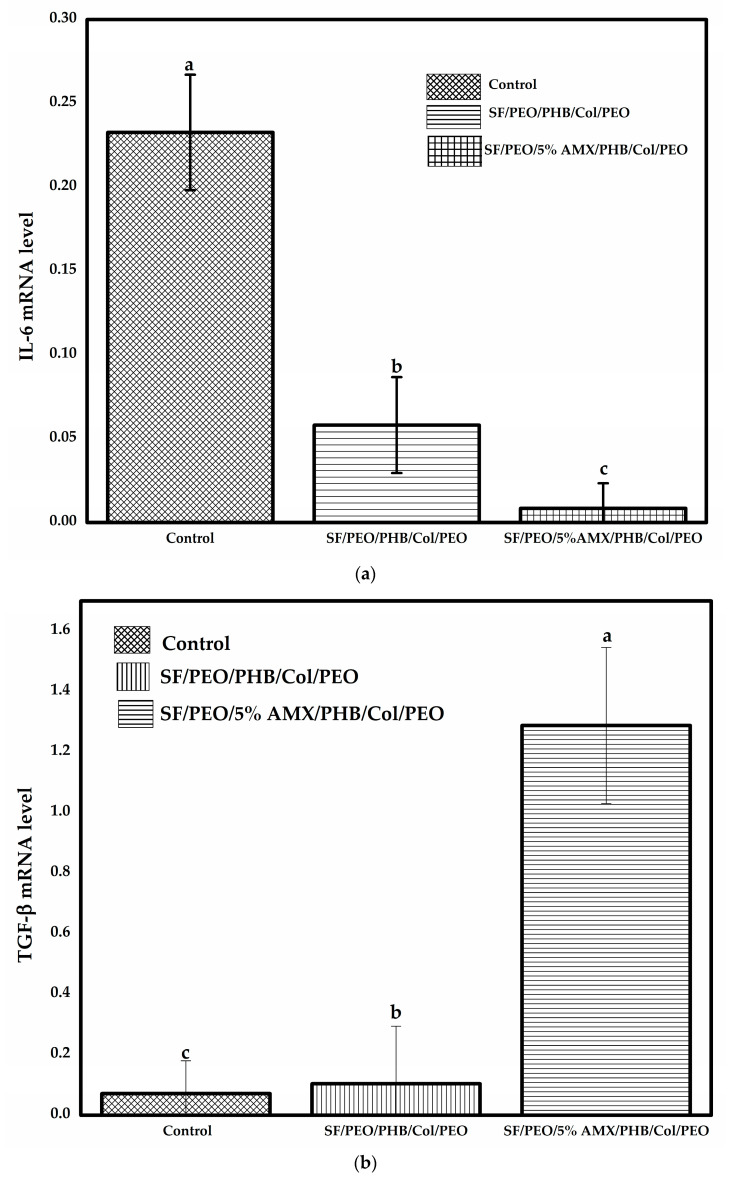
Gene expression levels related to wound healing on day 14 post-wounding. (**a**) IL-6 and (**b**) TGF-β. Different letters a, b, and c express the statistically significant difference for a *p*-value ≤ 0.05.

The wound repair process comprises successive steps that initiate immediately after an injury and develop into a complete skin reconstruction. The process involves the coordinated action of multiple cell types (resident and circulating cells homing to the wound site), the ECM, and soluble mediators named cytokines [83]. The wound repair process begins with coagulation and hemostasis, which halt bleeding and initiate the cellular response. In addition, the activated platelets within the clot release significant growth factors and cytokines that stimulate the resident cells to initiate angiogenesis, re-epithelialization, and connective tissue restoration. Any perturbation to these steps leads to chronic wounds [84]. IL-6 is a potent immunologic mediator that plays a fundamental role in inflammation, representing a substantial physiological phase in normal wound healing. The study of the expression of IL-6 in wound healing revealed that its expression was upregulated following injury in human, animal, and in vitro models. It was reported that overexpression of IL-6 following injury stimulates the production of various pro-inflammatory cytokines from existing cells, including stromal cells, keratinocytes, endothelial cells, and macrophages. In combination with the expression of anti-inflammatory mediators, macrophages are reconfigured from M1 pro-inflammatory to M2 tissue repairing [85].The delay in this conversion step results in delayed healing, an increased risk of infection, and scar formation [86]. The results revealed that both blank SF/PEO/PHB/COL/PEO and SF/PEO/5%AMX/PHB/COL/PEO nanofibrous scaffold-treated wounds significantly inhibited (*p* < 0.05) the expression level of IL-6 gene compared to sterile-gauze-treated wounds (Figure 9a). The faster a wound heals, the lower the expression of inflammatory factors, and vice versa. This significant decrease in IL-6 gene expression can be attributed to the potent anti-inflammatory and immune-enhancing properties of the SF/COL nanofibrous scaffold. A hybrid SF/COL nanofibrous scaffold has been reported to hasten wound closure and tissue restoration in vivo [82,87]. Moreover, the level of IL-6 gene expression was lower in wounds treated with SF/PEO/5%AMX/PHB/COL/PEO nanofibrous scaffolds compared to wounds treated with blank SF/PEO/PHB/COL/PEO nanofibrous scaffolds. Through incorporation into the SF/PEO/PHB/COL/PEO nanofibrous scaffold, AMX is slowly released at the wound site, preventing bacterial infections and accelerating the healing process, contributing to the superior anti-inflammatory activity of AMX-loaded nanofibers. Furthermore, the released AMX is readily engulfed at the inflammation site by immune cells such as macrophages and produces localized effects on the wound [78,88].

Growth factors are crucial for proper wound repair. TGF-β is a type of pluripotent cytokine that is produced primarily by macrophages and plays a significant role in cell proliferation and migration, immune regulation, apoptosis, and inflammatory response. It is also involved in all healing processes, such as the stimulation offibroblast generation, differentiation of fibroblasts into myofibroblasts, and synthesis of collagen I and II [89,90]. In addition, TGF-β1 can stimulate the healing process via activation of angiogenesis and augment the ECM production via both fibroblast and differentiated myofibroblast [62]. As shown in (Figure 9b), the expression level of the TGF-β1 gene in the group topically treated with SF/PEO/5%AMX/PHB/COL/PEO nanofibrous scaffold was significantly enhanced (*p* < 0.05) in comparison to SF/PEO/PHB/COL/PEO nanofibrous and sterile-gauze-treated group. This enhanced expression level in the case of treatment with AMX incorporated SF/PEO/5%AMX/PHB/COL/PEO nanofibrous scaffold might be due to induction in the formation of contractile bundles of normal fibroblasts [91].

A significant increase in both wound closure size and wound healing percentage was previously reported by Abo El-Ela, F.I, and his colleagues after topical application of AMX loaded into Layered Double Hydroxide (LDH) nanocomposite (AMOX/LDH) compared to the non-treated and control group [78]. They attributed this result to the excellent penetration capacity of the LDH nanocomposites carrying the antimicrobial agent that accelerate the healing process via preventing infections. Moreover, a higher expression level for the TGF-β1 gene was recorded for groups treated with blank nanofiber (collagen/silk fibroin nanofibers) compared to the sterile-gauze-treated group. Due to the activation of macrophages to produce chemotactic growth factors (GF), angiogenesis, and fibroblast proliferation via upregulation of TGF-β1, bFGF (primary fibroblast growth factor), and α-SMA (α-small muscle actin) genes, topical application of collagen isolated from tilapia skin resulted in optimal and standard cutaneous wound healing in the rat model [62]. It was previously reported that diabetic wounds in mice treated with silk fibroin/poly-(L-lactide-co-caprolactone) nanofiber scaffolds had a significantly higher TGF-β1 gene expression level than wounds in untreated mice.

## 4. Conclusions

Wound dressing nanofibrous scaffolds fabricated from natural components have garnered significant interest as promising wound dressings. In the current research, three layers of a biocompatible, multifunctional, antibacterial, biopolymer-based, hierarchically structured nanofibrous scaffold were fabricated. The bottom and top layers contain hydrophilic silk fibroin from natural SF and fish skin COL for accelerated wound healing, interspersed with a middle layer of hydrophobic PHB containing AMX as an antibacterial drug. The fabricated hierarchically structured nanofibrous scaffold elucidated sustained in vitro AMX release, good mechanical properties, high cytocompatibility, and enhanced antimicrobial effect. This hierarchically structured nanofibrous scaffold improves in vivo wound healing in rats, alleviating inflammation and increasing tissue epithelization. Therefore, it can be concluded that this nanofibrous scaffold can be utilized as an effective wound dressing.

## Figures and Tables

**Table 1:** Mean tensile strength and mean elongation at break values of SF/PEO, COL/PEO, SF/PEO/PHB/COL/PEO, 5%, and 10% AMX incorporated nanofibrous scaffolds.

Sample Name	Mean Tensile Strength (N/mm^2^)	Mean Elongation at Break (%)
SF/PEO	9.19 ± 0.05	2.70 ± 0.3
COL/PEO	27.77 ± 0.03	1.11 ± 0.2
SF/PEO/PHB/COL/PEO	4.16 ± 0.08	0.60 ± 0.5
SF/PEO/5%AMX/PHB/COL/PEO	35.17 ± 0.06	1.84 ± 1.1
SF/PEO/10%AMX/PHB/COL/PEO	86.13 ± 0.03	2.50 ± 1.2

**Table 2:** Wound closure rates of nanofiber-treated groups and untreated positive control.

Wound Diameter (mm) (Mean ± SDM) and % of Wound Closure (W.C.)		Observation Day				
		ZERO-DAY	Day 3	Day 7	Day 10	Day 14
Positive Control (sterile gauze)	Diameter	187.28 ± 1.98	170.26 ± 4.87	131.87 ± 3.17	62.94 ± 0.90	25.21 ± 6.32
	% W.C	0	9.09	29.59	66.39	86.54
SF/PEO/PHB/COL/PEO	Diameter	187.42 ± 1.40	142.10 ± 2.79	73.46 ± 4.40	22.72 ± 2.81	2.96 ± 2.75
	% W.C	0	24.18	60.80	87.88	98.42
SF/PEO/5%AMX/PHB/COL/PEO	Diameter	185.08 ± 3.30	85.64 ± 6.44	54.05 ± 4.60	7.26 ± 0.67	0.68 ± 0.64
	% W.C	0	53.73	70.80	96.08	99.63

## Data Availability

All data are available in the manuscript and supplementary materials.

## Supplementary Materials

The following supporting information can be downloaded at: https://www.mdpi.com/article/10.3390/pharmaceutics15051518/s1, Figure S1: Average diameter distribution of SF/PEO. Figure S2: Average diameter distribution of PHB. Figure S3: Average diameter distribution of Col/PEO. Figure S4: SEM cross-sectional photograph of SF/PEO/PHB/Col/PEO.
