# Supplementary material for: Topically Applied Biopolymer-Based Tri-Layered Hierarchically Structured Nanofibrous Scaffold with a Self-Pumping Effect for Accelerated Full-Thickness Wound Healing in a Rat Model
Source: Pharmaceutics. 2023 May 17;15(5):1518. doi: 10.3390/pharmaceutics15051518 (PMC10223825; doi:10.3390/pharmaceutics15051518)
Supplement: Supplementary file 1 [file pharmaceutics-15-01518-s001.zip › pharmaceutics-2142607-supplementary.pdf]

pharmaceutics

MDPI

Supplementary materials

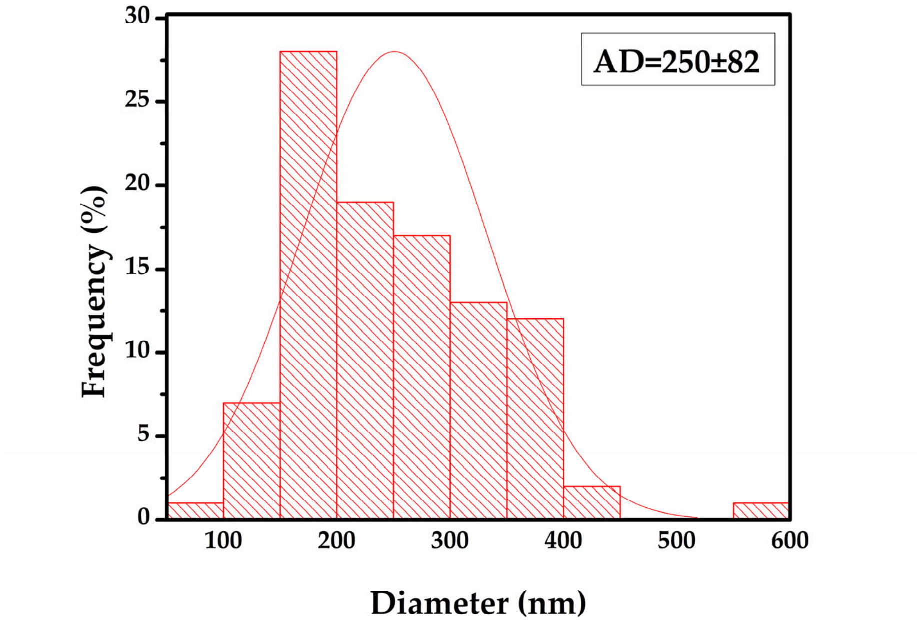

Figure S1. Average diameter distribution of SF/PEO.

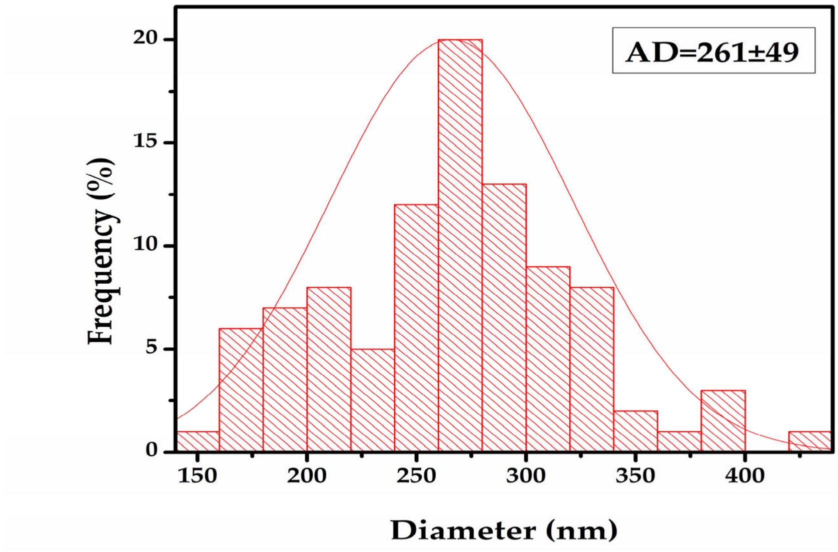

Figure S2. Average diameter distribution of PHB.

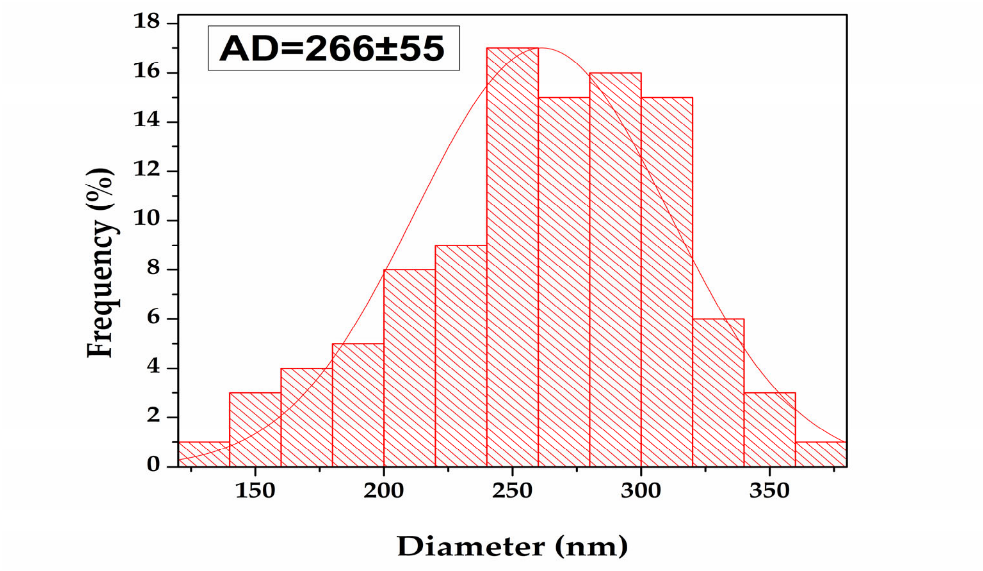

Figure S3. Average diameter distribution of Col/PEO.

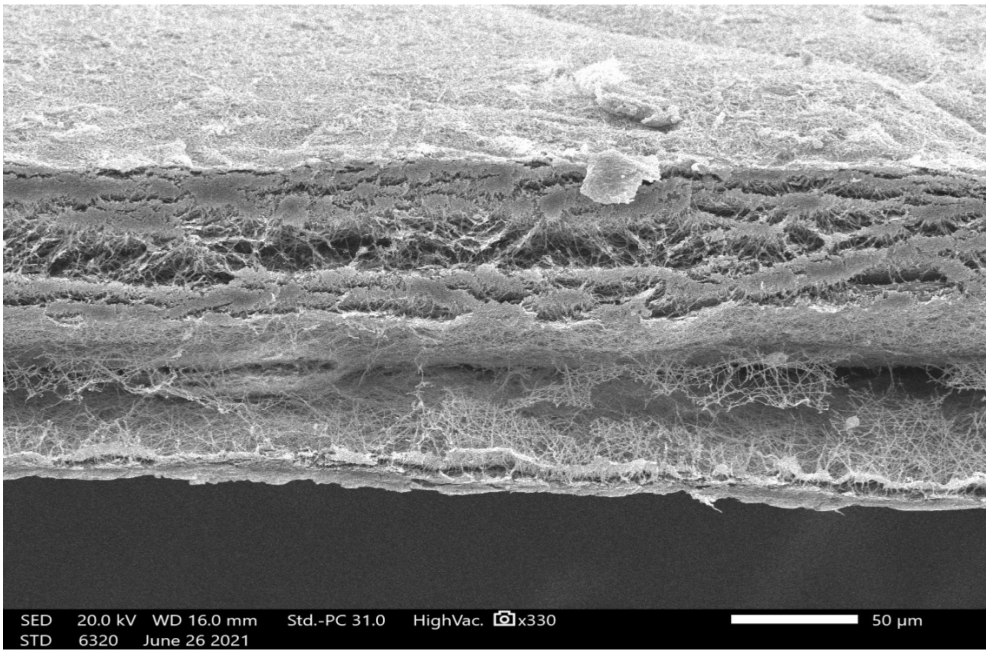

Figure S4. SEM cross sectional photograph of SF/PEO/PHB/Col/PEO.
